# Atomistic Simulations of Defect Production in Monolayer and Bulk Hexagonal Boron Nitride under Low- and High-Fluence Ion Irradiation

**DOI:** 10.3390/nano11051214

**Published:** 2021-05-04

**Authors:** Sadegh Ghaderzadeh, Silvan Kretschmer, Mahdi Ghorbani-Asl, Gregor Hlawacek, Arkady V. Krasheninnikov

**Affiliations:** 1 Institute of Ion Beam Physics and Materials Research, Helmholtz-Zentrum Dresden-Rossendorf, 01328 Dresden, Germany; s.kretschmer@hzdr.de (S.K.); mahdi.ghorbani@hzdr.de (M.G.-A.); g.hlawacek@hzdr.de (G.H.); 2 Department of Applied Physics, Aalto University, 00076 Aalto, Finland

**Keywords:** two-dimensional materials, h-BN, ion irradiation, photo-emitters, defects, atomistic simulations

## Abstract

Controlled production of defects in hexagonal boron nitride (h-BN) through ion irradiation has recently been demonstrated to be an effective tool for adding new functionalities to this material, such as single-photon generation, and for developing optical quantum applications. Using analytical potential molecular dynamics, we study the mechanisms of vacancy creation in single- and multi-layer h-BN under low- and high-fluence ion irradiation. Our results quantify the densities of defects produced by noble gas ions in a wide range of ion energies and elucidate the types and distribution of defects in the target. The simulation data can directly be used to guide the experiment aimed at the creation of defects of particular types in h-BN targets for single-photon emission, spin-selective optical transitions and other applications by using beams of energetic ions.

## 1. Introduction

Defects in solids, which are frequently portrayed as culprits only capable of deteriorating materials’ properties, have recently been, if not fully ’acquitted’, referred to positively due to their important role in various quantum phenomena and related possible applications. Specifically, intrinsic and extrinsic defects in semiconductors and insulators including silicon carbide [1,2,3], diamond [4,5], and transition metal dichalcogenides (TMDs) [6,7] have been shown to perform at room-temperature as quantum emitters and sensors.

Hexagonal boron-nitride (h-BN) [8,9] has also received a considerable amount of attention in the context of defect-mediated optical response and potential applications in quantum technologies [10,11,12,13]. This material with a rather wide bandgap of 5.95 eV [14] has high mechanical and temperature stability and is usually used as a substrate due to its insulating properties and small surface roughness. Moreover, it can be exfoliated down to a single h-BN sheet, representing together with graphene truly two-dimensional (2D) materials. In agreement with the results of first-principles calculations [11,15,16,17], h-BN, when enriched with defects, has been shown to exhibit single-photon emission [10,11,18,19,20] and spin-selective optical transitions [21,22], which can be used in optical quantum computing [23].

Ion irradiation is routinely used nowadays to create defects in various solid targets with the desired concentration. Moreover, by choosing proper ion species and adjusting ion energies [24] and charge states [25], one can control the types of defects and their distribution in the sample. With regard to the creation and use of defects in quantum applications, ion beam mediated engineering of defects has indeed been demonstrated to be an effective tool to produce quantum emitters [3,10,13].

However, the efficient use of this approach requires careful choice of the irradiation parameters. The response of mono-layer h-BN on metal substrates to heavy-ion irradiation has been studied for a narrow range of ion energies (low-energy limit [26,27]), along with the ions in MeV range [28,29] or in high charge states [30], but the systematic experimental studies of the response of single and few-layer h-BN to ion irradiation are scarce, and the comprehensive picture of the behavior of this system under ion bombardment cannot be drawn based solely on the experimental data. We note that in a wider context, understanding the response of h-BN to irradiation is also important for the use of this material in various radiation-related applications, such as neutron and particle detectors and scintillators [9,28,31]. To this end, atomistic computer simulations have been demonstrated to be a useful tool to get microscopic insights into defect production and choose the range of parameters optimal for a specific task. At the same time, impacts of energetic ions onto h-BN have received rather small amount of attention from theory. Irradiation of h-BN with oxygen ions [13] in a narrow energy range was addressed, and the response of single-layer h-BN to noble gas ion irradiation was also investigated [32]. Impacts of Xe ions onto multi-layer h-BN have also been simulated [33]. However, a Tersoff-type potential by Albe and Möller for h-BN [34] was used in the works [32,33], while the accuracy of this potential for defect simulations has not been systematically tested.

Here, we employ a recently developed extended Tersoff potential (ExTeP) [35] to study the production of defects in both mono- and multi-layer h-BN under noble gas ion irradiation in a wide range of ion energies. The potential has been carefully benchmarked against the results of density-functional theory (DFT) calculations of defect formation energies in h-BN. Moreover, we investigate not only the single-impact, but also the high-fluence limit.

## 2. Computational Details

To get microscopic insights into the interaction of energetic inert gas ions with monolayer and multilayer h-BN, we performed analytical potential molecular dynamics (MD) simulations using the LAMMPS computational package [36] as well as ab-initio molecular dynamics implemented in the VASP code [37,38] as detailed below. We stress that in the analytical potential MD, ions can be treated only as neutral atoms. The neglect of charge transfer during the collisions and also electronic excitations certainly affect the outcomes of ion impacts in semiconductors and insulators, that is the types of the produced defects and their concentrations. However, for the range of ion energies we consider, the ballistic energy transfer governs defect production, so that the results should be at least qualitatively correct, as demonstrated previously for a wide range of non-metallic materials, see, for example, [39] for an overview. We also note that even DFT-based Born-Oppenheimer MD cannot describe correctly the evolution of the electronic subsystem during ion impacts, and the Ehrenfest dynamics combined with time-dependent DFT should be used, which is computationally too expensive for systematic studies of effects of ion irradiation on a material. We refer the readers to Ref. [39] for an overview of the methods used in the simulations of irradiation effects in solids and a discussion of their accuracy and applicability.

### 2.1. Simulation Setup

**Figure 1:**
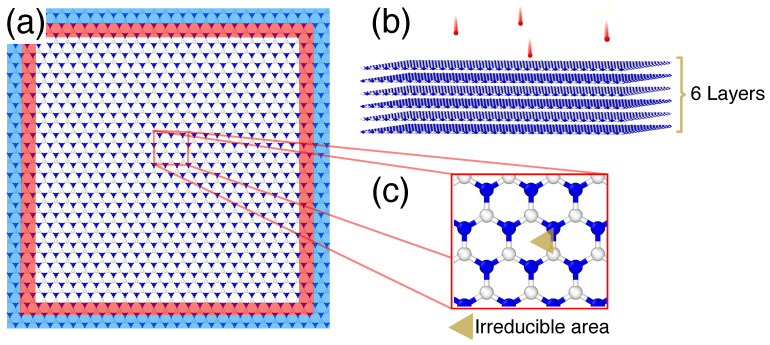
Simulation setup for monolayer (**a**) and multilayer (**b**) h-BN target. The outermost atoms (blue) were kept fixed during the irradiation simulations to avoid target displacement. The kinetic energies of the atoms were scaled in the areas indicated by red using Berendsen thermostat to describe energy dissipation at the borders of the simulation cell. (**c**) The irreducible area used in the single-ion-impact limit.

The simulation setups for the ion irradiation of mono- and multilayer h-BN are shown in Figure 1a,b. We stress that free-standing sheets were considered, which corresponds to the material deposited on a TEM grid or suspended over a trench in the substrate. The atomic positions in the structures were initially optimized using the analytical potentials described below. The impacts of the inert gas atoms onto the system were then simulated using MD. The initial (absolute) temperature of the system was zero and periodic boundary conditions were employed.

Different behaviors of atoms were defined and applied to different parts of the system for better performance. The outermost atoms were kept fixed during the simulations, as schematically shown in Figure 1a, to avoid displacement of the system as a whole under ion impacts. The velocities of the atoms were scaled down in the border areas using the Berendsen thermostat, mimicking the energy dissipation in an infinitely large sheet. Three thermostat regions near the supercell boundaries were defined, each of which, with a different dissipating power in order to effectively dissipate the outgoing energy waves. Free MD was carried out in the central region, where the impacts occur. The central region has a reasonable distance from the thermostats and boundaries, and the impact-induced cascades do not directly interfer with the thermostat and fixed regions. The simulation setups for single-ion vs. high-fluence irradiation, were, however, slightly different, as described below.

#### 2.1.1. Irradiation Setup: Single-Ion Impact Limit

The term single-ion impact simulation is used to refer to the low-fluence limit in which ions interact with the pristine system. The projectiles we studied had incident energies in the range of 1 eV–1 MeV. Following the impact, the system was allowed to evolve in time until the thermodynamic equilibrium was reached. The target temperature was then quenched to 0 K. In order to account for all possible impacts, the concept of the irreducible area in the h-BN primitive cell was employed. A total number of 512 impacts per irreducible area for each set of parameters (atom-type, energy) were simulated and the presented results were averaged over them.

#### 2.1.2. Irradiation Setup: High-Fluence Irradiation

The computational demand rises with increasing the size and complexity of the target from mono- to multi-layer h-BN, and the projectiles from single to high-fluence irradiation. Thus, for the more computationally demanding parts, an energy range of 50–200 eV was considered.

The following fluences for single charged ions were modelled: $1\times 10^{14}$ ions/cm${}^{2}$, $5\times 10^{14}$ ions/cm${}^{2}$ and $1\times 10^{15}$ ions/cm${}^{2}$. High-fluence irradiation was simulated as a sequence of impacts onto random regions of the central part of the target, that is the damage from the previous impacts was preserved. The system then evolved with time and it was slowly quenched to zero temperature before the arrival of the next incident ion. In this setup, the damage that sequential impacts caused to the target accumulates. This means that the entire simulation of high-fluence irradiation is a sequential and continuous process, and resembles the behavior of the system under real irradiation conditions.

### 2.2. Interatomic Potentials

The interactions between boron and nitrogen atoms were modelled by an extended Tersoff potential (ExTeP) [35]. This potential accurately describes the most common low-energy B, N, and BN structures and yields quantitatively correct trends in the bonding as a function of coordination. The vacancy formation energies reported by the developers of ExTeP show a good agreement with the ab-initio results. The developers of ExTeP further argue that none of the existing potentials for BN, including the one used in a previous study of h-BN irradiation [32], are meant to describe various defects that occur in the irradiated materials. The Ziegler-Biersack-Littmark (ZBL) [40] potential was used for the interaction of noble-gas ions with the target atoms.

In addition to the previous tests, the reliability of the ExTeP potential in describing atomic displacements was further evaluated by the comparison with the results of ab-initio Born-Oppenheimer MD (DFT-MD) calculations (Table 1). The DFT-MD calculations were carried out as implemented in the VASP computational package to assess the displacement threshold of B/N atoms from a monolayer h-BN with 48 B + 48 N atoms in the supercell. A plane-wave cutoff of 300 eV was used and the Brillouin zone was sampled with a 5×5×1 k-mesh grid. A micro canonical ensemble (NVE) was simulated and the MD time steps were set to 0.2 fs. We stress that by ‘displacement threshold’ we mean the minimum ion energy required to displace the atom, not the minimum kinetic energy which should be assigned to the recoil atom to displace it, as when, for example, assessing the damage under electron irradiation [41,42].

Although the absolute values of the displacement thresholds calculated by ExTeP are higher (by 5–25%) than the values obtained by VASP, which is likely a result of complex electronic interactions between the projectile and the target at different velocities that are not captured explicitly by the analytical potential, the boron/nitrogen threshold ratios agree well. This suggests that the number of created boron vacancies relative to the number of nitrogen vacancies can be reliably interpreted and hence the employed approach provides an overall correct behaviour.

### 2.3. Data Analysis

The vacancies created in the system were counted and identified using a modified version of the Wigner-Seitz analysis. The vacancies are categorized into three groups—single, double and complex vacancies. ‘Single’ refers to a missing boron or nitrogen atom, and when not directly specified, the single vacancy refers to the sum of B and N vacancies. ‘Double’ refers to a missing B-N pair in the target, and term ‘complex’ is used for any bigger vacancies in the system with more than two missing atoms. The complex vacancies can also be interpreted as pores created in the system by the ions.

## 3. Results and Discussion

The number of defects produced in h-BN sheets under irradiation is known to depend on ion fluence, incident energy, projectile mass (i.e., chemical element) and the target thickness. The influence of each of these parameters was assessed independently and/or combined to get a clearer insight into the defect production process, as described in the separate sections.

### 3.1. Monolayer h-BN

#### 3.1.1. Single-Ion Irradiation

The average number of vacancies, which is the best descriptor of the damage creation process, is calculated as the ratio of the total number of vacancies to the number of impact events. Note that although this is not the probability for a specific type of defect to appear (it can obviously exceed unity), these calculations provide an estimate of single B or N vacancy abundance at different incident energies for various projectile species.

**Figure 2:**
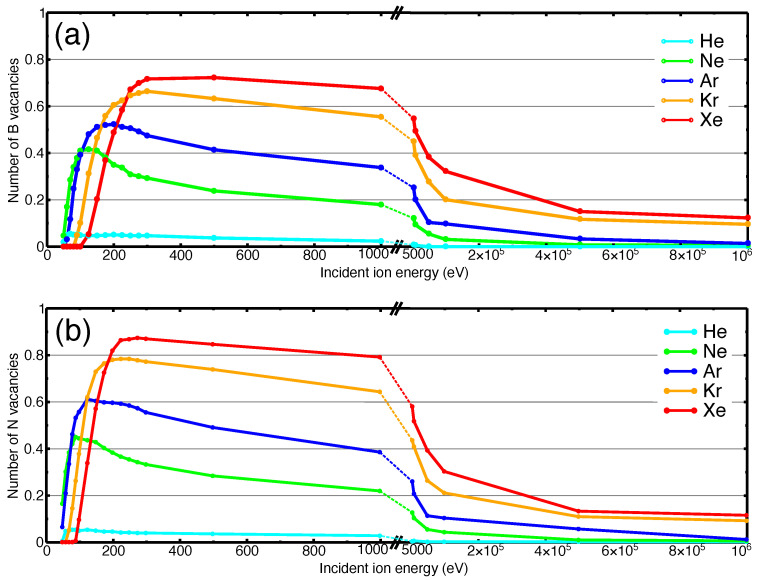
Average number of (**a**) single B vacancies, and (**b**) single N vacancies in h-BN monolayer created under irradiation with various inert gas ions. The apparent ‘kink’ at ion energies about 5 keV is just a visual effect originating from the transition from linear to logarithmic scale.

Fixing the incident angle to normal, the rates of vacancy creation in monolayer h-BN under irradiation of He, Ne, Ar, Kr and Xe ions over a wide energy range were assessed. The average numbers of B and N vacancies are shown in Figure 2a,b.

Due to the fact that in the process of single-ion irradiation at normal incidence, the incident ions predominantly sputter only one target atom, the number of sputtered atoms are approximately the same as the number of induced vacancies. Therefore, sputtering-yield as a function of the incident energy would practically give the same plot as presented in Figure 2. Tilting the incident angle away from normal changes this situation and there may be a higher sputtering yield as previously demonstrated for single ion [43,44] and cluster irradiation [45] of 2D materials.

As evident from Figure 2, there is an energy threshold (minimum ion energy) for vacancy creation, followed by a rapid increase in the average number of produced defects as incident ion energy increases. The probability of creating vacancies saturates at energies above 200 eV and eventually a gradual decay can be seen. Further increase in energy does not give rise to the formation of more vacancies. Such behaviour at high energies is a result of smaller impact cross-section of nuclear stopping power, as demonstrated previously [33,46]. Overall, such a dependence with a clear maximum on defect production probability vs. ion energy curve is typical for 2D materials [47], for example, graphene [48], which has a very similar atomic structure.

The numbers of produced vacancies at intermediate and high energies differ substantially depending on the mass of the ion. More massive ions have a higher probability of producing vacancies. However, the order of the curves at low incident energies is affected by mass-comparability between projectile and the target-atoms (that is by the kinematic factor, which describes the energy transfer in a head-on binary collision of two particles) which defines the amount of energy transferred to the recoil atom in this energy regime. The higher probabilities for creating N single vacancies in Figure 2b, as compared to B single vacancies Figure 2a, is in agreement with the lower formation energies of N vacancies in the h-BN structures [35].

#### 3.1.2. Simulations of High-Fluence Irradiation

Multiple projectile impacts onto the same area in the target occur under high fluence irradiation, and because the response of the defective system to irradiation can be different from that of the pristine material, the accumulation of defects and the associated changes in the atomic structure must be taken into account to adequately describe the production of defects. In our simulations, each impact cascade was allowed to evolve until the energy brought in by the energetic ion is redistributed over the system, then the temperature was quenched to 0 K before the arrival of the next incident ion. By changing the number of the incident ions per supercell, we studied the effects of the fluence. The high-fluence simulations were performed for mono- and multiple-layers of h-BN, each of which is presented in different sections. The irradiation fluences considered in this work are $1\times 10^{14}$ ions/cm${}^{2}$, $5\times 10^{14}$ ions/cm${}^{2}$ and $1\times 10^{15}$ ions/cm${}^{2}$.

Increasing fluence should naturally give rise to a higher density of single vacancies. Moreover, multiple ion impacts onto the same area should give rise to double or complex vacancies. One should also note that the impacts of incident ions onto an already damaged region can produce more damage, as atoms in this region are not as strongly bound as in the pristine system.

In the following sections, we focus on lower/intermediate energies, that is, 50–200 eV, considering that the projectiles at very low incident energies do not penetrate the target, and at high energy, on the other hand, the probability for defect production drops, as seen from Figure 2, and ion ranges increase, so that much larger supercells should be considered to model the behavior of the system, which is associated with high computational costs.

**Figure 3:**
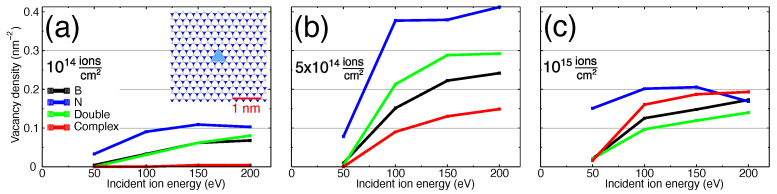
Comparison of the concentration of vacancies in h-BN sheet after irradiation with Ar ions with three different fluences as functions of ion energy: (**a**) $1\times 10^{14}$, (**b**) $5\times 10^{14}$ and (**c**) $1\times 10^{15}$ ions/cm${}^{2}$. The inset in panel (**a**) illustrates the average area for single defect when defect density is 0.1 nm${}^{-2}$.

**Figure 4:**
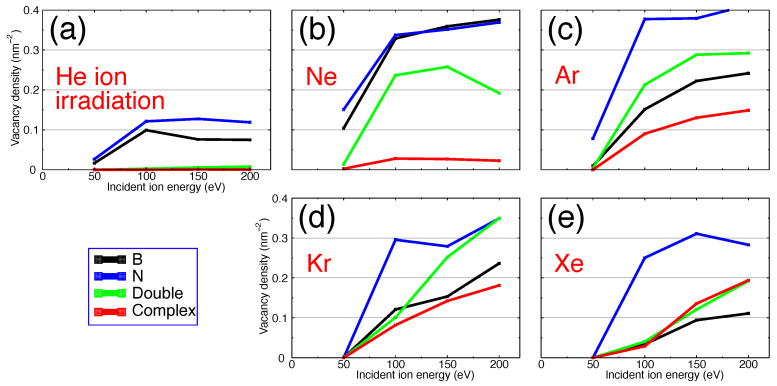
Densities of vacancies in single layer h-BN after irradiation with a fluence of $5\times 10^{14}$ ions/cm${}^{2}$ as functions of ion energies for various inert gas ions. Panels (**a**–**e**) show irradiation with He, Ne, Ar, Kr and Xe ions, respectively.

The concentrations of vacancies (areal vacancy density) in single-layer h-BN produced upon Ar ion irradiation with three different fluences are presented in Figure 3 as functions of ion energies. As evident from Figure 3, the irradiation fluence of $5\times 10^{14}$ ions/cm${}^{2}$ may be considered as an upper limit before complex vacancies become abundant and the target layer noticeably amorphizes or even holes are formed. On the other hand, fluences below $5\times 10^{14}$ ions/cm${}^{2}$ must produce predominantly single vacancies. Therefore using fluences in the range of between $1\times 10^{14}$ ions/cm${}^{2}$ and $5\times 10^{14}$ ions/cm${}^{2}$ is expected to give rise to isolated yet abundant single and double vacancies. In the following sections we will use a fluence of $5\times 10^{14}$ ions/cm${}^{2}$ as a characteristic fluence for the controlled single and double vacancy creation without destroying the target. The density of produced vacancies under this irradiation fluence for different projectiles is shown in Figure 4.

As evident from Figure 3 and Figure 4, the concentration of N vacancies is almost always higher than that of B-vacancies and multivacancies, which correlates with the higher vacancy formation energies obtained in DFT calculations and the values given by the developers of ExTep potential [35]. It is also in line with our ab-initio MD calculations of displacement thresholds (Table 1).

The results presented in Figure 4 indicate that double (green curve) and complex (red curve) vacancies are not produced under He ion irradiation, and the total number of single vacancies created by He is lower than that produced by heavier projectiles. Ne ions generate a high number of single and double vacancies in the absence of a noticeable number of complex defects. Heavier ions produce all types of vacancies. A clear increase in the concentration of complex vacancies from He to Xe, panels (a) to (e), is visible. Xe produces a smaller number of single and double vacancies, in favour of creating more complex vacancies.

**Figure 5:**
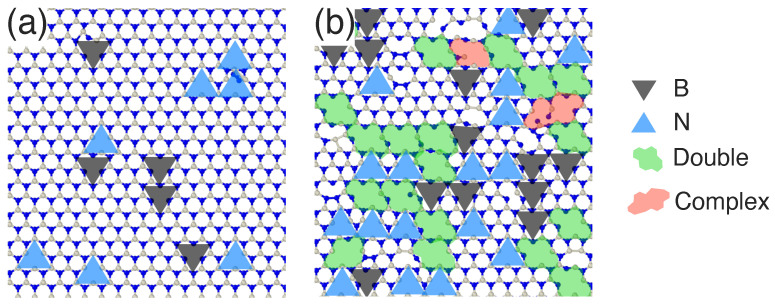
Atomic structure of h-BN sheet after irradiation with Ne ions having energy of (**a**) 50 eV and (**b**) 150 eV.

Examples of the atomic structure of the h-BN target after irradiation with Ne ions having different energies are shown in Figure 5. Two energies were selected, namely 50 and 150 eV, and the fluence was $5\times 10^{14}$ ions/cm${}^{2}$. It is clear from the figure that for the same fluence, at low energy only single vacancies were created, while at the higher energy a mixture of different vacancy types was found after the irradiation.

**Figure 6:**
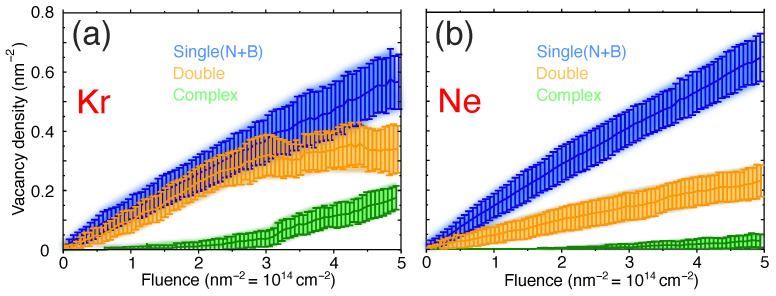
Accumulation of different types of vacancies during ion irradiation with different ions. Single (N + B) is the sum of single N and single B vacancies. The projectiles in this plot are: (**a**) Kr ions with 200 eV energy, and (**b**) Ne ions with 100 eV energy. The standard-deviations and averages for each incident projectile are shown with solid lines and vertical bars respectively. Note that to facilitate the comparison between defect densities and fluences, we present fluence in nm${}^{-2}$, not cm${}^{-2}$).

The vacancy accumulation during irradiation, that is the increase in vacancy density with fluence, separating a high/low fraction of complex vacancies, is shown in Figure 6. After irradiation with a fluence of 3 nm${}^{-2}$ (to facilitate the comparison between defect densities and fluences we present fluence in nm${}^{-2}$, not cm${}^{-2}$), a mixture of single, double and complex vacancies are produced. Two different situations are illustrated—Vacancy creation under 200 eV Kr ions, where eventually complex and double vacancies are more abundant, is presented in Figure 6a. Irradiation with 100 eV Ne ions where more single and double vacancies were produced is shown in Figure 6b. One must note that the maximum fluence is relatively low in our simulations, but if the fluence is increased, defect density should saturate to certain values. This is due to the fact that the target is already damaged at this stage and subsequent impacts will not generate new vacancies, but rather create more complex vacancies and amorphize the target, which may even result in a drop in the number of single vacancies in the system.

### 3.2. Multilayer h-BN

To get insight into vacancy creation in multilayer h-BN, irradiation simulations for 6-layers of this material were carried out. The number of layers is chosen in such a way that the penetration depths of the projectiles at studied incident energies, that is, 50–200 eV, is smaller than the thickness of the system. Each data point on the plots is an average over 10 independent simulations, a fewer number as compared to what we used in the simulations for mono-layers, which provides a reasonable compromise between the accuracy and computational costs, which are obviously much higher for the multi-layer system.

**Figure 7:**
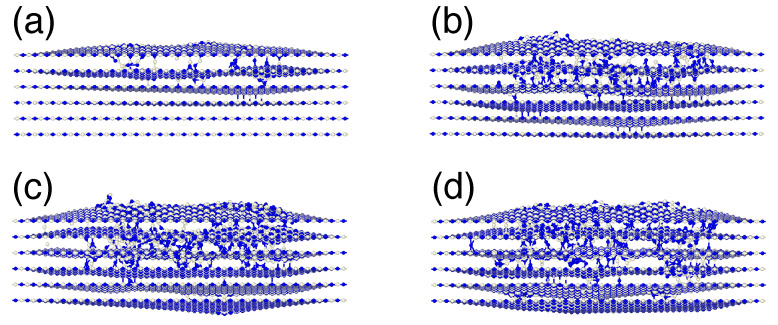
Typical atomic structures of multi-layer h-BN after irradiation with Ne ions with incident energies of 50 (**a**), 100 (**b**), 150 (**c**) and 200 (**d**) eV, respectively.

The typical atomic structures after impacts of Ne ions with four different energies are shown in Figure 7. The fluence is the same and is equal to 5 $\times \;10^{14}$ ions/cm${}^{2}$. It is evident that contrary to free-standing single-layers of h-BN, irradiation gives rise to the formation of not only vacancies, but also interstitial-type defects located between h-BN layers, similar to the defects studied previously using DFT calculations [49].

#### 3.2.1. Depth Distribution of Irradiation-Induced Defects in Multilayer h-BN Targets

Here we analyse the distribution of irradiation-induced vacancies in different h-BN layers. We consider various inert-gas projectiles and an intermediate fluence of $5\times 10^{14}$ cm${}^{-2}$.

**Figure 8:**
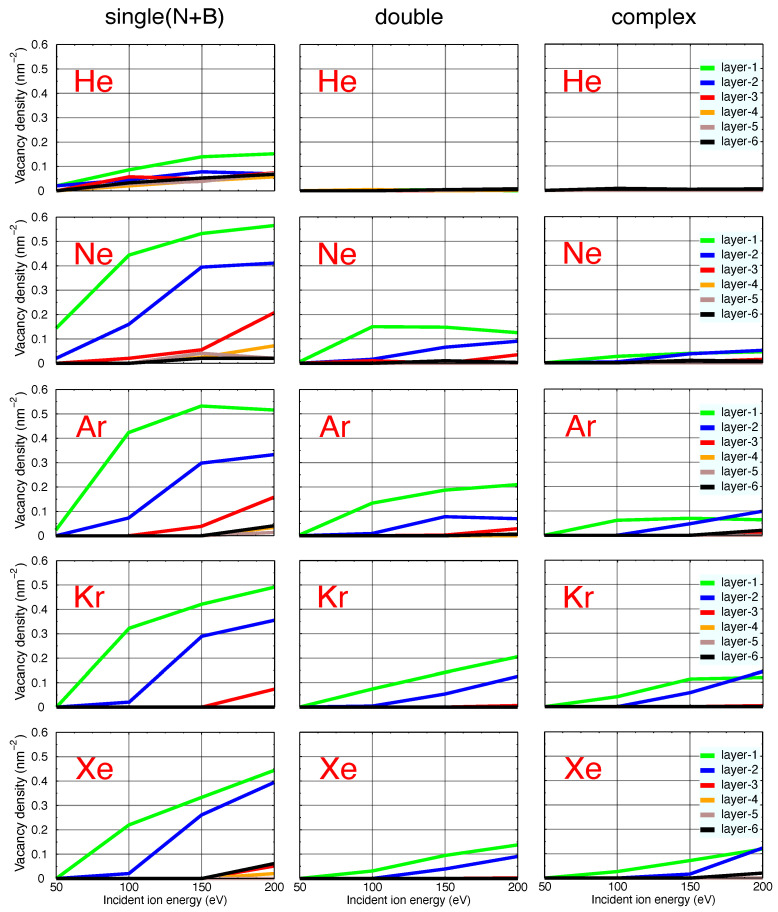
Densities of vacancies created in different layers upon irradiation with various inert gas ions. The rows of plots correspond to projectiles of the same type, while the columns show vacancies of the type. Single (N + B) is the sum of single N and single B vacancies. Different h-BN layers are color-coded. To facilitate the comparison of the effects of different ions, the scale is the same in all the plots.

The results of our calculations are shown in Figure 8, where the curves corresponding to defects in different h-BN layers are color-coded. Plots in different columns stand for different types of vacancies. To facilitate the comparison of the effects of different ions, the scale is the same in all the plots. It is evident that lighter projectiles, as compared to their heavier counterparts, have a larger penetration depth, and therefore, a higher chance of creating vacancies in the deeper layers. The relation between ion mass and defect size is also as expected, that is heavier ions create larger defects. He ions for instance do not produce double and complex vacancies at these intermediate energies. However, due to their small weight and therefore long range, they create single vacancies in all—including the deep—layers. As a consequence, the single vacancies induced by the He projectiles, although low in number, are more homogeneously distributed in the h-BN layers as compared to all the other species. Heavier incident ions deposit their kinetic energies in the top layers and in the case of Kr and Xe are more or less unable to produce vacancies beyond the second layer. This is true until the kinetic energies of the heavy projectiles exceed the regime shown here. In this case and based on the results for monolayer h-BN at high energies (Figure 2), the probability for creating vacancies in the top layers drops. These ions penetrate deeper, slow down and eventually deposit their energies in deeper layers. One can conclude from Figure 8 that heavier ions are better candidates for vacancy creation in top h-BN layers under the studied energy regime, without causing noticeable damage to the lower layers. On the other hand, lighter projectiles show a better performance in creating more homogeneously distributed single vacancies in a larger number of layers while the presence of complex vacancies is still low. Figure 8 also indicates that the topmost layer contains the highest density of vacancies at intermediate energy regimes. The absence of complex defects for He makes He ion irradiation preferable for the creation of single-photon emitters based on single vacancies [20,21].

**Figure 9:**
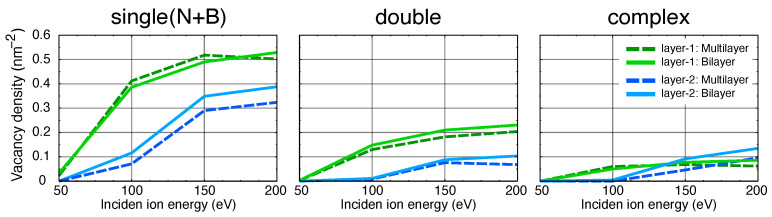
Comparison of defect production in a bilayer h-BN and the top two layers of the multilayer target under high fluence ($5\times 10^{14}$ ions/cm${}^{2}$) Argon ion irradiation. The solid lines stand for the bilayer system and the dashed lines represent the multilayer target, and the types of the vacancies are shown above each plot.

In order to understand how the production of defects in the top layers is affected by the presence of the underlying layers, we carried out ion irradiation simulations for bilayer h-BN and compared the results to those for the multi-layer system. The results for high fluence of $5\times 10^{14}$ ions/cm${}^{2}$ and Argon ion are presented in Figure 9. Although the density of single-vacancies in the top-most layer (green color) is slightly affected by reducing the h-BN layers to two, the second layer (blue color) has undergone a more noticeable change and the induced single vacancies are clearly increased in the studied energy regime, as compared to the multilayer system. This is due to the lack of any material underneath the second layer in the bilayer target, whose atoms have therefore more freedom to escape from the system and leave vacancies behind. At the same time, the density of double and complex vacancies, although being small, are also overall increased in the bilayer system. Therefore, one can conclude that in a free-standing bilayer h-BN more vacancies are produced than in the multilayer system under the studied irradiation fluence and incident energy regime. For supported materials, this effect will likely be diminished by the substrate [50], as the substrate will play the role of the third layer and also facilitate in-situ defect annealing [51]. However, for lighter ions (e.g., He) and higher energies, the presence of substrate can increase the number of produced defects, as shown previously for other 2D materials [43].

#### 3.2.2. Depth-Dependent Energy Threshold for Vacancy Creation in Multilayer h-BN

**Figure 10:**
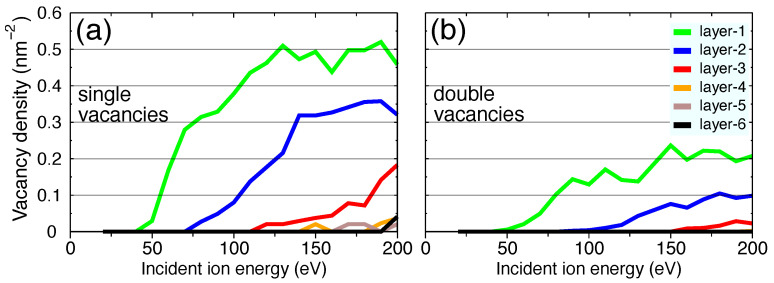
Ion energies for creation of vacancies in h-BN layers. (**a**) Single vacancies; (**b**) double vacancies. The color-code indicates different h-BN layers as shown in the legend.

In order to get a rough estimate of how many layers are damaged upon irradiation, it is desirable to assess what energy the ion should have to produce vacancies in different h-BN layers. Therefore, we chose Ar as the projectile and conducted a set of simulations starting from low energies. The vacancy densities in various layers as functions of ion energies are shown in Figure 10, and the results for different layers are color-coded.

Figure 10 shows that the lowest ion energy threshold for vacancy creation is ∼40 eV, which is close to the value obtained for isolated h-BN monolayers (Table 1). Interestingly, this plot indicates that the energy thresholds for deeper layers are increased by steps of approximately 40 eV. This result comes from the fact that the h-BN layers are identical and that in the low energy limit the ions almost always lose energy in the topmost layer. The ions create vacancies/pores and only then reach the deeper layers. There is certainly a small portion of the ions which channel deep, and defects can be created by the primary recoil atoms. For instance a few atoms of the second layer were tracked down below the 4th layer. Nevertheless, statistically a big number of the ions create defects in the topmost layers first.

## 4. Conclusions

We performed analytical potential MD simulations aimed at getting microscopic insight into the defect production in mono- and multi-layer h-BN under noble gas ion irradiation at various fluences, over a wide range of incident energies. Our results show that under normal incidence more defects are produced in thin h-BN targets with increasing incident ion energy, but up to the nuclear stopping power maximum, after which the vacancy yield decreases again. We note that the number of defects will start growing up at much higher ion energies due to energy deposition into electronic excitations followed by their conversion to defects, as h-BN is an insulator. In this work we also provide datasets for other relevant factors, like ion mass and fluence, which also affect the production of defects. Comparing different irradiation fluences at a fixed incident ion energy however, reveals a strong influence of the fluence on the formation of vacancies, and the vacancy type, namely single, double or complex vacancies. High irradiation fluences such as $1\times 10^{15}$ ions/cm${}^{2}$ create a noticeable amount of complex vacancies, leading to amorphization and ultimately complete destruction of the h-BN target. At the same time, low fluences such as $1\times 10^{14}$ ions/cm${}^{2}$, are not sufficient to generate complex vacancies and pores, and also the density of induced single/double vacancies is low. Varying irradiation fluence between $10^{14}$ and $5\times 10^{14}$ ions/cm${}^{2}$ affects the ratio between single and double vacancies. Our results for different types of vacancies show that the most abundant vacancies are N-vacancies.

Simulations of the irradiation of multilayer h-BN indicate that at low/intermediate energies (i.e., 50–200 eV) vacancies are mostly produced in the topmost layer, and that deeper layers contain fewer defects, as expected. Our results, however, give precise information on the defect distribution in the h-BN target. While heavier incident ions deposit their kinetic energy in the top layers, the lighter projectiles penetrate deeper and are capable of creating vacancies in deeper regions. He ions at this energy regime produce a homogeneous distribution of single vacancies throughout h-BN layers in the absence of double and complex vacancies. This capability makes He a good candidate for generation of low-density single vacancies with homogeneous vacancy distribution in the target. Overall our results quantify defect concentration and defect distribution in single- and multi-layer h-BN targets and can directly be used to guide the experiment aimed at the creation of defects of particular types in h-BN targets for quantum applications.

## Figures and Tables

**Table 1:** Comparison of ion energies (displacement thresholds, $T_{d}$) required to displace boron/nitrogen atoms using the Born-Oppenheimer DFT MD (DFT-MD) and the employed analytical potential (AP).

Projectile	AP ${\mathrm{Td}}_{B}$ (eV)	AP ${\mathrm{Td}}_{N}$ (eV)	DFT-MD ${\mathrm{Td}}_{B}$ (eV)	DFT-MD ${\mathrm{Td}}_{N}$ (eV)	AP ${\mathrm{Td}}_{B}/{\mathrm{Td}}_{N}$	DFT-MD ${\mathrm{Td}}_{B}/{\mathrm{Td}}_{N}$
He	40.49	41.63	32	40	0.8	0.97
Ne	40.09	35.71	31.5	30.0	1.05	1.12
Ar	51.30	43.30	38.5	35.0	1.10	1.18

## Data Availability

The data supporting the results reported in this study are fully presented in this article.

## Abbreviations

The following abbreviations are used in this manuscript:

hBN	Hexagonal Boron-Nitride
TMD	Transition Metal Dichalcogenide
2D	Two Dimensional
MD	Molecular Dynamics
DFT	Density Functional Theory
